# Gun-Shot Wound

**Published:** 1859-11

**Authors:** J. S. Weatherly

**Affiliations:** Montgomery, Alabama


### ARTICLE II.

*Gun-Shot Wound.* By J. S. WEATHERLY, M. D., Montgomery, Alabama.

I was requested, on the 18th of September, 1858, to visit a man thirty miles below Montgomery, on the Pensacola R. R., the messenger at the same time informing me that he had been shot with a Repeater early in the morning, and that he was in a critical situation, and would probably be dead before my arrival. I accordingly got ready, and taking the cars at 12 o'clock at night, I arrived at my patient's house at 2 o'clock, A. M., being about sixteen hours since he had received the wound. I found a tall, muscular man, of nervo-sanguine temperament. He was not suffering much pain, but seemed nervous and restless—had not slept any. His pulse was 135 per minute, and he was feeling faint and very weak. On examining the wound, I found that a ball had entered the epigastrium, a very little below, and to the right of, the ensiform cartilage. Introducing a probe, I found the direction of the wound to be downwards, and to the right; but as the relations of the parts were changed, and it was not prudent to place him in the position that he was when he received the wound, I contented myself with very little probing, soon satisfying myself that I could not reach the ball. I examined the whole surface of his body, but could find no trace of the ball. I was informed that soon after receiving the wound, he fainted and fell to the ground; that he vomited about a half pint of blood, and had an involuntary operation from his bowels. His friends thought him dead, but he finally rallied, and was carried home, a distance of one mile. The person who shot him was standing within a house, and my patient was just stepping into the door; he was consequently a little below his antagonist. The instrument used was a Colt's Repeater, and the ball was conical. I was at a loss to tell what had become of the ball, as I could detect no trace of it, either externally or internally. From the direction of the traces of the ball, as far as I was able to explore, it must have penetrated the stomach; and the vomiting blood would also lead to the conclusion that that organ was wounded. If it had pursued the same direction that it started, it would have penetrated the liver; and it was in that organ that I thought it most probable that the ball had lodged.

My prognosis was unfavorable, for I thought that, if he did not die from the immediate effects of the wound, he would be carried off by peritonitis or hepatitis, or both.—Believing in the antiphlogistic powers of opium, I determined to give him the full benefit of that powerful remedy. I accordingly prescribed a large dose for him, to be taken immediately, and that he was to be kept under the influence of it all the time. I also had his abdomen covered with lint, saturated with tinct. arnica and water. He was to have pillets of ice, and to be nourished with cold rice water. I saw him again in three days, and found him doing well; his pulse down to one hundred; very little signs of fever; no suppuration about the wound. He was to be kept on the same treatment. His bowels were moved by an enema on the seventh day.

On the fifteenth day, he suffered some neuralgic pains in the region of the liver—this, however, soon subsided. About this time I ordered his diet to be increased a little, and his opium to be diminished. On the twentieth day, I pronounced him out of danger. The wound was entirely well. There had been no peritonitis, and no suppuration, the whole tract healing by the first intention. The principal features in the treatment consisted in keeping him under the influence of opium, and perfectly quiet. The Arnica, I have no doubt, also had some effect in keeping down inflammation. But what could have become of the ball? The man is as well now as he ever was, and has never felt any pain that would indicate the position of the ball. Could a large ball become incisted in the liver without producing more uneasiness than this man suffered? or, could it have lodged within the cavity of the stomach and passed out thro' the bowels? I was so far from him that I could not examine his discharges; tho' his brother, who is a very intelligent man, could detect nothing resembling a ball. Altogether, it was an interesting case to me, and if you think it sufficiently interesting for a place in your Journal, you will please insert it.

